# A randomized controlled trial of customized adherence enhancement (CAE-E): study protocol for a hybrid effectiveness-implementation project

**DOI:** 10.1186/s13063-022-06517-0

**Published:** 2022-08-04

**Authors:** Jennifer B. Levin, Farren Briggs, Carol Blixen, Mark Bauer, Douglas Einstadter, Jeffrey M. Albert, Celeste Weise, Nicole Woods, Edna Fuentes-Casiano, Kristin A. Cassidy, Julie Rentsch, Kaylee Sarna, Martha Sajatovic

**Affiliations:** 1 Department of Psychiatry, Case Western Reserve University School of Medicine, Cleveland, OH USA; 2 Neurological and Behavioral Outcomes Center, University Hospitals Cleveland Medical Center, 10524 Euclid Ave. 7th floor, Cleveland, OH 44106 USA; 3 Department of Psychiatry, University Hospitals Cleveland Medical Center, Cleveland, OH USA; 4 Department of Population and Quantitative Health Sciences, Case Western Reserve University School of Medicine, Cleveland, OH USA; 5 Department of Psychiatry, Harvard Medical School, Boston, MA USA; 6 Department of Medicine, MetroHealth System, Cleveland, OH USA

**Keywords:** Medication adherence, Bipolar disorder, Manic-depressive disorder, Randomized controlled trial, Telehealth

## Abstract

**Background:**

Mood-stabilizing medications are a cornerstone of treatment for people with bipolar disorder, though approximately half of these individuals are poorly adherent with their medication, leading to negative and even severe health consequences. While a variety of approaches can lead to some improvement in medication adherence, there is no single approach that has superior adherence enhancement and limited data on how these approaches can be implemented in clinical settings. Existing data have shown an increasing need for virtual delivery of care and interactive telemedicine interventions may be effective in improving adherence to long-term medication.

**Methods:**

Customized adherence enhancement (CAE) is a brief, practical bipolar-specific approach that identifies and targets individual patient adherence barriers for intervention using a flexibly administered modular format that can be delivered via telehealth communications. CAE is comprised of up to four standard treatment modules including Psychoeducation, Communication with Providers, Medication Routines, and Modified Motivational Interviewing. Participants will attend assigned module sessions with an interventionist based on their reasons for non-adherence and will be assessed for adherence, functioning, bipolar symptoms, and health resource use across a 12-month period. Qualitative and quantitative data will also be collected to assess barriers and facilitators to CAE implementation and reach and adoption of CAE among clinicians in the community.

**Discussion:**

The proposed study addresses the need for practical adherence interventions that are effective, flexible, and designed to adapt to different settings and patients. By focusing on a high-risk, vulnerable group of people with bipolar disorder, and refining an evidence-based approach that will integrate into workflow of public-sector care and community mental health clinics, there is substantial potential for improving bipolar medication adherence and overall health outcomes on a broad level.

**Trial registration:**

The study was registered on ClinicalTrials.gov NCT04622150 on November 9, 2020.

**Supplementary Information:**

The online version contains supplementary material available at 10.1186/s13063-022-06517-0.

## Introduction

### Background and rationale

A cornerstone of treatment uniformly recommended by guidelines on treatment for individuals with bipolar disorder (BD) [1] is mood-stabilizing medication such as lithium, anticonvulsants, or second-generation antipsychotic drugs [2–5]. However, approximately 50% of individuals with BD are poorly adherent with their medication [6–9] often leading to severe and negative consequences [10–16]. Poor adherence in BD is associated with poor recovery and high relapse [17]. Gonzalez-Pinto [18] reported a 5.2-fold increased suicide rate in BD patients with poor adherence compared to adherent BD patients [18]. Other reports note substantially increased costs for individuals with poor vs. good adherence [19, 20]. Since poor adherence in BD is a robust predictor of mood relapse [21], adherence promotion is a logical target for BD treatment and recovery efforts.

A review on BD adherence research studies conducted by this study team suggests that while a variety of approaches including psychoeducation, motivational interviewing, financial incentives, and cognitive behavioral treatment can all be of some benefit in improving medication adherence in BD, there is no single approach that appears to have superior adherence promotion efficacy, and there are very limited data on how adherence enhancement approaches can be implemented in clinical settings where most people with BD receive care [1]. Two literature reviews on the topic of BD adherence highlighted the fact that interventional approaches need to be flexible and designed to adapt to different settings and patients [22, 23].

The COVID-19 global pandemic has underscored the need to have interventions that are amenable to virtual delivery [24]. However, a recent systematic literature review by Basit and colleagues on telemedicine interventions for pharmacological adherence in persons with bipolar disorder, schizophrenia, or major depressive disorder [25] found only a limited number of trials (*N*=17) and only two studies that included individuals with BD. Another recent review on eHealth interventions in individuals with chronic health conditions generally found that a majority of interactive eHealth interventions are effective in improving adherence to long-term medication [26]. Intervention strategies that improve patient’s treatment involvement and their medication management skills appear most promising [26].

Customized adherence enhancement (CAE) is a brief, practical BD-specific approach that identifies individual patient adherence barriers and then targets these areas for intervention using a flexibly administered modular format [27, 28]. A prospective, 6-month, randomized controlled trial (RCT) of CAE vs. a rigorous BD-specific educational control in poorly adherent patients found that both adherence and functional status were improved in CAE vs control [29]. Remarkably, the benefits of CAE occurred in individuals living with BD, on average, over two decades, with extensive psychiatric comorbidity and who were more than 40% non-adherent with BD medication at study enrollment.

While promising, the original efficacy RCT was limited by the fact that it was performed in an academic medical center, did not make use of existing web/text messaging technology, and did not address potential challenges to scale up in standard clinical settings. This Type 1 hybrid effectiveness-implementation project will adapt CAE for use in community/public-sector care settings, test effectiveness in high-risk, poorly adherent individuals in these settings, and gather evidence on barriers and facilitators to implementation of the intervention to inform subsequent scale-up and spread. The project will examine putative mechanistic engagement targets suggested by previous work and include implementation elements that will inform future dissemination.

### Trial design

The proposed 5-year project uses a Type 1 hybrid effectiveness-implementation design intended to accelerate translation while at the same time providing a valid estimate of potential clinical effectiveness and target engagement evaluation specific to adherence behaviors. The Type 1 hybrid design incorporates formal assessment of barriers and facilitators to implementation into the traditional clinical RCT design, in order to speed the process of future scale-up and spread of the intervention. In such designs, model-guided mixed methods analyses are conducted across multiple levels (patient, provider, system, community) that can be used for subsequent intervention adaptation and implementation efforts [30].

In Phase 1 (months 1–6), we will refine the CAE intervention for content and process guided by stakeholders (patients/family, clinicians, administrators). The stakeholder advisory board (SAB) will guide modest refinement of the program including supplemental CAE materials to meet diverse stakeholder needs and help guide strategies to integrate CAE into clinical workflows at a community mental health clinic (CMHC) and a safety-net county healthcare system. Evaluation of CAE refinement will be guided by the integrated Promoting Action on Research Implementation in Health Services (i-PARIHS) framework which holds that successful implementation is a function of characteristics of the innovation, the recipients (patients and providers), the inner and the outer contexts (setting /environment), and facilitation support [31].

In Phase 2 (months 7–60), we will conduct a prospective, randomized effectiveness-implementation superiority trial of technology-facilitated CAE vs. enhanced treatment as usual (eTAU). In total, 190 participants will be randomized at baseline on a 1:1 basis to receive either CAE (*N* = 95) or eTAU (*N*=95). CAE is a brief adjunct to standard mental health care. The primary outcome is adherence measured by the Tablet Routines Questionnaire (TRQ) and validated with electronic pill monitoring via eCAP^TM^. Secondary outcomes include functioning, health resource use, and BD symptoms. All study participants will be followed for a 12-month period. We will assess barriers and facilitators to CAE implementation using both qualitative and quantitative methods. To evaluate patient-level mechanistic targets, we will explore the effects of personal adherence barriers (insufficient or inaccurate BD knowledge, unstable routines, poor treatment alliance, and substance use) and adherence facilitators (care engagement, patient satisfaction, ease of access). Finally, we will assess reach and adoption of CAE among clinicians (referral counts) and health resource use including outpatient care, emergency room visits, and hospitalizations.

In considering the appropriate comparator to CAE in this effectiveness trial, we chose an approach that is generalizable to standard clinical care. Outcome assessment in most adherence trials typically involve monitoring of pill-taking behavior, and it has been noted in previous studies, including this study team’s preliminary work, that adherence monitoring itself is likely to improve adherence [32]. Thus, adding adherence monitoring via electronic pill caps (eCAPs^TM^) and text messaging to standard care is an appropriate and generalizable comparator.

### Objectives

The proposed study has 4 specific aims: (1) to refine the CAE intervention guided by stakeholders (patients/family, clinicians, administrators); (2) to test the effectiveness of technology-facilitated CAE vs. eTAU using a prospective, 2-site RCT; (3) to test the effects of CAE vs. eTAU on functional status in poorly adherent individuals with BD; and (4) to identify barriers and facilitators to CAE implementation in order to inform subsequent scale-up and spread using qualitative methods and guided by an implementation conceptual model. As with Aim 1, Aim 4 evaluation will be guided by the i-PARIHS framework.

Additional exploratory analyses will assess whether changes in patient-level adherence barriers and facilitators mediate the treatment effects on adherence. Finally, the project will evaluate on-site (outpatient visits, no-show rates) and off-site (emergency department visits, hospitalizations) health resource use to help characterize relative value and inform future sustainability efforts. An over-arching goal of this project will be to provide a curriculum-driven adherence enhancement approach that can be implemented in public-sector care settings and which can improve outcomes for the most vulnerable groups of people with BD. Given the substantial negative effects of poor adherence on BD outcomes, the project has high public health significance.

## Methods

The study methods follow the SPIRIT reporting guidelines [33] and are verified with a completed SPIRIT Checklist.

### Participants

Study coordinators will enroll 190 RCT participants, up to 12 SAB members, and 10 providers/administrators with whom they will conduct qualitative interviews. Referrals will be sought from clinicians at two public-sector mental health centers, a CMHC, and a public urban safety-net health system. CAE pilot and RCT enrollment suggests that a substantial proportion of participants will be racial minorities [34–36].

Patients will be recruited by a variety of methods at the CMHC and safety-net system where the participants receive their care. Recruitment will be done by oral request to patients being seen for regularly scheduled clinical visits. Requests for participation may be initiated by a clinical staff member, by a research associate on site, or by patients themselves. Psychiatrists and other clinicians will be asked to discuss this project with eligible individuals and to coordinate introductions of study staff. Electronic health records may be queried to determine a list of potential participants at each site and Institutional Review Board (IRB)-approved advertising may also be utilized at these sites. Recruitment strategies will be discussed at regularly scheduled meetings with CMHC/safety-net system staff and the study team. All referred individuals with BD will be considered for possible inclusion in the study.

### Inclusion and exclusion criteria

The RCT participants will include adults ages 18 to 89 who have a diagnosis of BD Type I or Type II for at least a two-year duration and as determined by the Structured Clinical Interview for DSM-5 Research Version (SCID-5-RV) [37] at screening. RCT participants must present with a BPRS score of at least 36 at screening. They must also have received treatment with at least one evidence-based medication to stabilize mood for at least 6 months (lithium, anticonvulsant, or antipsychotic mood stabilizer) and be poorly adherent with prescribed BD medication treatments (missing ≥ 20% of prescribed BD medication within past week or month). All participants must be able to participate in psychiatric interviews and give written informed consent. Those who are at high immediate risk for harm to self or others or are part of the study’s SAB will be excluded from the RCT population.

The SAB will include adults ages 18 to 89 with BD who receive their care in safety-net care systems and/or a CMHC *or* family members of individuals with BD *or* clinicians or administrators who practice at safety-net care systems or CMHCs. Those who are unable/unwilling to give written, informed consent to study participation will be excluded. The provider/administrator participants in qualitative interviews will include adults ages 18 to 89 who are clinicians or administrators at the CMHC or safety-net system and exclude those who are unable/unwilling to give written, informed consent to study participation.

### Study setting

The research team will conduct research from the PI’s research offices, which are located at an urban academic medical center. The team will also conduct some study procedures at the CMHC and safety-net system where participants are recruited. Research Electronic Data Capture (REDCap) will be used for secure data entry and storage [38]. The consent process may be conducted either in person or via IRB-approved videoconferencing. Other study procedures and visits may also be conducted in person or via videoconferencing, phone, and/or via REDCap survey (individual link emailed directly to the participant) in the event an in-person visit is not possible. The study intervention will be delivered remotely via videoconferencing, or if that is not possible, by phone or in person at the CMHC or safety-net system. In addition, SAB meetings will be conducted remotely via videoconference.

### Phase 1: intervention adaptation and guidance

Building upon strong existing partnerships between members of the study team and public-sector mental healthcare partners in Northeast Ohio, the study team will obtain input from the SAB to refine the CAE intervention content to meet the needs of patients and other stakeholders in CMHCs and safety-net care systems and suggest how CAE might be best incorporated into clinical workflow. The SAB will be composed of up to 12 relevant stakeholders including 4 individuals with BD who receive their care in safety-net care systems and/or a CMHC, 2 family members of individuals with BD, 4 clinicians, and 2 administrators who practice in these locations. Consistent with i-PARIHS framework, stakeholders will represent intervention recipients as well as the inner context and outer context of implementation efforts (see Table 1). To expand input from non-research stakeholders, we will formalize the structure of the SAB such that a SAB Chair is identified by SAB nomination and/or vote on an annual basis. The SAB Chair will meet with members of the study team to provide his/her recommendations to the team and will serve as an additional interface between community stakeholders and the study team.

**Table 1:** Stakeholder Advisory Board themes/discussions mapped onto the i-PARIHS framework

i-PARIHS domain	Qualitative themes
Innovation	Perceived value of remotely delivered CAE sessions Perceived CAE module alignment with patient needs
Recipients	Patient and clinician perceptions of benefit vs. burden of CAE
Inner and outer context	Clinician perceptions of how CAE does/does not integrate with site workflow Health system administration perceptions of relative value of CAE vs. training and implementation burden SAB perceptions on how CAE may align/not align with broader healthcare priorities
Facilitation	Mental health interventionist perceptions of CAE training, comfort with intervention

Basic demographic and information about experience with bipolar disorder (i.e., lived vs. professional experience and duration of experience) will be collected from the SAB participants. There will be 3 video conference calls during the first 6 months of the project using Zoom or a similar videoconferencing platform. In the first call, SAB members will review the CAE curriculum and identify content areas that they feel may need to be edited or added. The study team members will then make these modifications to the CAE intervention manual. It is expected that at least some of the added content will be appropriate to compile a supplemental content “toolkit” that can be used as needed based on patient needs. In the second meeting, SAB members will review the revised CAE content and make any additional suggestions. In the third SAB meeting, the SAB will be asked to identify strategies that will be helpful to integrating CAE into clinic workflow. SAB meetings will be audio/video recorded and assessed qualitatively to inform the intervention. After Phase 1 is concluded, the SAB will continue to meet twice annually for years 2–5. It is expected that SAB input and guidance will continue to assist with optimal implementation and effectiveness of the project including maximizing recruitment and retention of participants and clinician engagement and referrals.

### Phase 2: implementation of interventions

Both CAE and eTAU are brief adjuncts to standard mental health treatment, and trial participants will continue to receive treatment with their regular mental health clinicians. Both arms will have remotely administered pill monitoring via an automated device called an eCAP^TM^ that records pill bottle openings to a secure, cloud-based database. CAE interventionists will be licensed social workers or mental health clinicians with equivalent training supervised by a PhD-level psychologist. A treatment manual for CAE has been developed which is intended for use by mental health staff in public-sector settings such as social workers and counselors. The manual provides explicit guidelines regarding how modules should be co-administered in single or multiple sessions to minimize redundancy and burden. The manual will be modified based on feedback from the SAB prior to implementation. Adapted content will include an optional modular content “toolkit” that will be appropriate for application in diverse settings and with diverse patients. An example of a toolkit item might be resources for helping individuals manage adherence with non-psychotropic medication prescribed for comorbid health conditions common among individuals with BD.

#### CAE

As in the efficacy CAE trial, CAE is comprised of a series of up to four treatment modules for which inclusion is determined based upon an individual’s reasons for non-adherence and personal adherence barriers. Adherence barriers are evaluated at the baseline evaluation with items from the Attitudes towards Mood Stabilizers Questionnaire (AMSQ) [39, 40] and Rating of Medication Influences (ROMI) [41]. The standardized modules are Psychoeducation, Communication with Providers, Medication Routines, and Motivational Enhancement Therapy (MET), which will be assigned based upon the same procedures and thresholds used in the CAE efficacy RCT [42]. An additional procedure will occur for assignment to Modified Motivational Enhancement Therapy (MET) interviewing. Namely, individuals with or without a substance use history will also be asked if they feel it might be helpful to participate in MET and those who answer affirmatively will receive MET. This may increase MET assignment for those who are reluctant to explicitly state that they use/abuse substances. CAE modules are derived from existing evidence-based approaches for patients with BD [43–48] and have been refined in the CAE pilots [34, 42, 49]. Each CAE module is broken down into units that can be combined with units from other CAE modules. It is intended that the units from all four CAE modules can be combined and integrated as needed to be delivered in no more than 4 sessions of 45–60 min each. Individuals who are only assigned to 1 or 2 modules will be given the descriptions of the other modules and asked if they would like to add any of them.

All CAE sessions will be conducted remotely by Zoom or a similar video conferencing application. Those who do not have internet access can join a video teleconference session from their respective clinical site or by phone with a printed copy of the materials. The first 4 sessions will be administered about 1 week apart and a final “booster” session to review previously delivered material will be 4 weeks after the completion of the 4 core sessions (total of approximately 5 sessions). CAE participants will set a behavioral goal for each session and will be given a small gift card as a reward for achieving that goal. There will be one Zoom/phone call check-in with the study interventionist that will occur in the 4-week time period between completion of 4 CAE core sessions and prior to the “booster” CAE session. As in the CAE RCT and pilots, it is expected that most (> 90% of participants) will have their assigned CAE modules implemented over 4 core sessions, depending on the number of modules assigned. Participants will be asked their preference of text messages or phone calls for reminding them of assessment appointments. Participants will be reminded of upcoming intervention sessions based on site procedures for appointment reminders. CAE participants will also receive monthly text messages to refill medications and put it in the eCAP^TM^ pill bottle as well as brief monthly general adherence promotion messages for the duration of the study.

1. Psychoeducation on Medication Treatments: Psychoeducation approaches BD as a biological disorder that can be managed by appropriate medication treatments in conjunction with behavioral coping strategies [50]. Psychoeducation has been noted to improve medication adherence [11, 51]. This module uses a modified Life Goals Program [43–46]. The module consists of 3 individual units including (1) basic information about BD, its neurobiological underpinnings, and information on mania and depression; (2) a focus on medication management, identifying the purpose of medication and reviewing good and bad effects of medication; and (3) following a discussion of functional impact of symptoms, the interventionist and individual with BD collaboratively develop a personal symptom profile for the individual’s own episodes of depression and mania as well as their early warning signs of impending relapse.
2. Communication with providers: Using principles from collaborative care [52–54], this module focuses on improving communication with providers from a patient-focused, patient-directed approach. Individuals with BD will be supported in examining and exploring key components of treatment planning with their provider including expectations for medication response and feared or experienced medication side effects. Key critical issues will include understanding of differential burden of medication-related effects and how these effects might be prioritized for discussion with a clinician. This 2-unit module also provides information on commonly utilized psychotropic agents.
3. Medication routines: Complex medication regimens may interfere with daily activities and adherence [55]. Using principles from interpersonal and social rhythm therapy for BD [48], this 2-unit module will focus on daily routine with respect to medication-taking and problem-solving regarding common barriers. This module will emphasize the use of prompts/reminders and self-monitoring/self-regulation to maximize and maintain adherence. A key activity in this module is a review of medication-taking patterns including examination of when, where, and how medications are taken. Accessible tools will be used to support medication taking including cellphone alarms, calendar reminders, or use of medication tracking and reminder apps.
4. Modified Motivational Enhancement Therapy (MET): MET is based on Motivational Interviewing, an evidence-based psychosocial intervention for individuals with dual diagnosis [47, 56–59]. This 2-unit module will help individuals understand the effects of substance abuse on their BD in general and on their adherence to medication specifically. Individuals will be encouraged to access personal motivation to change their substance use, making it more likely that they will be adherent to their medication regimen. The 2-unit module consists of an assessment of individual substance use/abuse followed by modified MET that addresses adherence specifically within the context of substance abuse.

#### eTAU

Individuals in eTAU will receive an eCAP^TM^ for their foundational BD medication. This, in itself, may improve medication adherence behaviors [32]. The eTAU participants will also receive monthly automated text messages to refill medications and fill their eCAP^TM^ as well as the same brief general adherence promotion messages received by the CAE group for the duration of the study.

### Engagement and retention

As this is a group of participants who may be particularly difficult to engage and retain in the study, there will be a number of measures taken to optimize retention. These include scheduling visits at maximally convenient times for participants, allowing for frequent breaks as needed during the procedures, hiring and training staff that will be flexible and attentive to the individual’s needs and frequent opportunities for questions and feedback. Those in the CAE group will receive items to support the adherence modules such as a pill-minder, water bottle with study logo, etc. of minimal monetary value but that will be perceived as a personal connection. Should there be challenges in meeting enrollment goals, the study team will request input from the SAB in procedures and processes that might optimize enrollment. Finally, participants in all treatment groups will be compensated for research visits and interviews.

### Assignment of interventions: randomization

Individuals will be randomized on a 1:1 basis to participate in either CAE or eTAU. Stratified randomization will be employed to ensure that equal numbers of CAE and eTAU patients are balanced with respect to BD type (I or II) and current substance abuse. Random effects will include a random intercept and possibly a random slope or other function of time as needed to suitably capture the within-person correlation structure (present/absent) at each site. The randomization list will be computer-generated by the biostatistician and integrated into a REDCap randomization project. Members of the study staff will not have access to the sequence prior to assignment and will utilize the REDCap project to randomly assign participants to study arm. Neither participants nor assessors will be blind to treatment condition. The lead trial statistician will be blinded for the analysis.

### Fidelity

Training of interventionists and measures to ensure fidelity to CAE will be similar to the procedures used in the CAE efficacy RCT for content/format for the duration of the study. To ensure generalizability of results to public-sector settings, each interventionist will devote only a portion of their overall time (0.25FTE) to the CAE intervention. After the initial training and throughout the study, CAE sessions will be recorded and 25% of the sessions (approximately 4 sessions every month) will be randomly selected and reviewed for fidelity by the senior interventionist or a fidelity rater trained by the senior interventionist. Fidelity assessment during the course of the study will evaluate CAE module-specific tasks on a Y/N or 0–10 scale. MET module fidelity assessment will include elements of the Motivational Interviewing Treatment Integrity (MITI) code [60]. Finally, CAE interventionists will participate in periodic teleconferences run by the senior interventionist to discuss implementation issues that may arise over time and in order to maintain standard approaches and minimize any potential variability.

### Assessments and outcomes

Table 2 shows the assessment measures and time-points, following the structure of a SPIRIT figure. Demographic and clinical variables measured at baseline will include age, gender, race/ethnicity, education, marital status, income, and health literacy [61]. Each participant will be assessed 6 times: at screening, baseline, 10 weeks, 6 months, 9 months, and 12 months. Data will be obtained from patient interviews. All assessments will be done by a rater trained to pre-established and documented reliability standards. It is expected that patient assessment will require approximately 120 min for the screening assessment and approximately 90 min for the follow-up assessments.

**Table 2:**
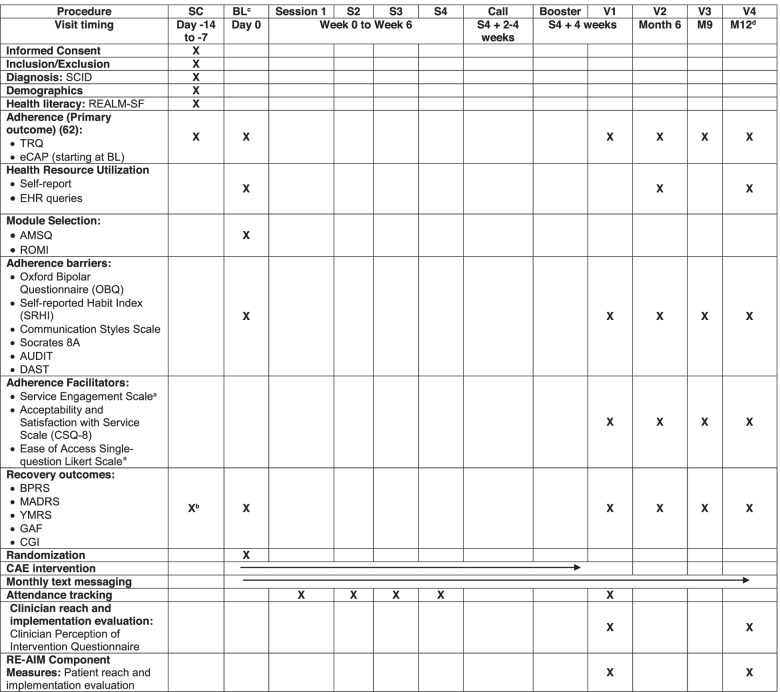
Phase 2 measures and schedule of events [62]

^a^Service Engagement Scale and Ease of Access question will only be administered at V1

^b^only BPRS and MADRS will be administered at Screen

^c^Baseline should ideally be completed 2 weeks after screen, but no less than 1 week after screen to allow for sufficient time for eCAPs to be used

^d^Individuals who terminate study prematurely will have termination visit study assessments done at the time point that termination actually occurs

#### Primary outcome

##### Adherence

Adherence is defined as the percentage of days with doses missed with a higher value indicating worse adherence. Adherence will be evaluated in two separate but complementary ways, the self-reported TRQ for the past week and past month and the eCAP^TM^. The TRQ [40] is reliable for use in BD and shows a high correlation with lithium levels [63]. Adherence will be assessed for each evidence-based BD maintenance medication (lithium, anticonvulsant, antipsychotic) prescribed for at least 3 months. For individuals who are on more than one medication, an average TRQ will be calculated. While it is true that the literature on measurement of adherence, including the team’s own work in this area, notes limitations with all methods of adherence assessment, both self-report and automated pill caps appear valid and practical for use in BD studies [1, 32].

*eCAPs*^*TM*^ *(Information Mediary Corporation, Ottawa, ON, Canada)*: Electronic monitoring of pill container openings is an important method of measuring adherence. Pill bottles equipped with eCAPs^TM^ are capable of storing a 90-day supply of one medication. Once the eCAP^TM^ is activated by the Certiscan® Secure Reader, the eCAPs^TM^ record each time they are opened. The data are uploaded to the cloud remotely by scanning the eCAP^TM^ via an app on the participant’s personal smartphone or by using the Certiscan® Secure Reader. This provides a precise, objective assessment of the timing of each dose and the patient’s pattern of medication-taking behavior. Although many patients with BD take multiple maintenance BD medications, study staff will monitor the BD drug missed most often (index drug). If more than one drug is dosed at the same frequency, the BD drug most recently added to the regimen will be the index drug. As-needed or “prn” drugs will not be monitored. In previous work by this study team, the correlation between a single “index” BD drug and all BD drugs was 0.95 providing support for measuring one medication as proxy for medication adherence [64]. The method of choosing an index drug also limits participant burden. To ensure that the data collected represent pill-taking behavior as accurately as possible, patients will be instructed to dispense doses of their monitored medication only from the eCAP^TM^-equipped bottle, remove only one dose at a time, and remove a dose only at the time that they plan to swallow the medication.

While just assessing adherence behavior may temporarily increase adherence (e.g., the Hawthorne effect), it is expected that any potential impact on adherence behavior will wane over the 12-month follow-up. A recent large clinical trial demonstrated limited long-term adherence advantage for passive medication aids (e.g., 7-day pill organizer, pill cap displaying time since last dose), a category into which eCAP^TM^ would fall [65]. Experience with eCAP^TM^ in a pilot BD and hypertension adherence study [66] conducted by members of the study team yielded substantially less missing data using the eCAP^TM^ than the Medication Event Monitoring System (MEMS) [32]. Adding an extra financial incentive for bringing in the eCAP^TM^ or scanning it using the free eCAP^TM^ app on the participant’s phone ($10 per visit) optimizes use of automated pill monitoring. Notably, in the previously mentioned iTAB-CV R21 project using eCAPs^TM^, 31/38 participants (81.6%) brought their eCAP^TM^ to all 3 study visits over a 6-month period [66]. To optimize generalizability, individuals who use medisets or pill-minders to manage multiple medications will not be excluded from the study.

Only one medication will be tracked via eCAP^TM^. All other medications can remain in the pill-minder or other medication tracking device. The medication is placed in the eCAP^TM^ for the first time by the participant while the research assistant observes either in person or via videoconferencing. Participants will be trained on the use of eCAP^TM^ and will have the opportunity to practice opening the cap using a sample bottle if in person or have it demonstrated via videoconferencing.

#### CAE engagement targets

CAE targets patient-level adherence barriers and facilitators.

##### Health literacy

The Rapid Estimate of Adult Literacy in Medicine - Revised (REALM-R) [61] is an 11-item instrument designed to rapidly screen patients for potential health literacy problems. The measure is quick, taking only a minute or two, and identifies the grade level of the patient if they read below the ninth-grade level.

##### Adherence barriers

The Oxford Bipolar Knowledge Questionnaire (OBQ), a 40-item self-report will be used to assess knowledge of BD management. The OBQ uses a 3-point Likert scale to assess BD knowledge domains [67]. Routines will be assessed using the Self-Report Habit Index (SRHI), a 12-item self-report measure of habit strength, and will be administered regarding the habit of taking medication [68]. Provider communication will be assessed via the Communication Styles Scale, a patient-rated measure of the impact of physician communication style on medication beliefs and adherence behavior in depressed patients [69]. Alcohol use will be evaluated using The Alcohol Use Disorders Identification Test – Self-Report Version (AUDIT), a 10-item measure used to screen for excessive alcohol use developed by the World Health Organization [70]. Drug use will be evaluated using the Drug Abuse Screening Test (DAST-10), a 10-item abbreviated version of the original 28-item DAST created to assess drug-related problems in the past year [71]. Motivation to reduce the use of substances will be assessed using the 19-item Stages of Change Readiness and Treatment Eagerness Scale (SOCRATES 8A) [72].

##### Adherence facilitators

Engagement will be measured with the Service Engagement Scale (SES) [73, 74] for individuals with mental health conditions. The SES has 14 items that are rated on a 4-point scale ranging from 0 (not at all) to 3 (most of the time). The four subscales refer to availability, collaboration, help seeking, and treatment engagement. Staring and colleagues [75] used the SES to evaluate individuals with psychotic disorders who received adherence therapy. The SES has shown good psychometric properties [74]. Data will also be collected on attendance and reasons for missed appointments. Intervention acceptability will be evaluated with the 8-item satisfaction with service Client Satisfaction Questionnaire (CSQ-8) adapted to reference CAE [76]. CAE intervention’s perceived ease of access will be assessed via a single-item Likert scale.

#### Recovery outcomes

##### Functioning

Functional status will be evaluated using the Global Assessment of Functioning (GAF) [77]. The GAF is a 100-point single-item scale which measures global functioning of psychiatric patients and is widely utilized in clinical studies involving patients with serious mental illness [78].

##### BD symptoms

While symptoms in patients with chronic serious mental illness do not consistently correlate with adherence or change in adherence [36, 75], symptom assessment is still an important indicator of treatment outcomes and can be used to help identify sub-groups of individuals who may or may not benefit from behavioral interventions. BD symptoms will be assessed using the Brief Psychiatric Rating Scale (BPRS) [79], the Young Mania Rating Scale (YMRS) [80], and the Montgomery Asberg Depression Rating Scale (MADRS) [81]. Global psychopathology will be measured with the Clinical Global Impressions (CGI) [82], a widely used scale which evaluates illness severity on a 1 to 7 point continuum. Severity of illness ratings on the CGI have reported reliability scores ranging from 0.41 to 0.66.

#### Process and qualitative evaluations

Consistent with a Type 1 effectiveness hybrid design [30], quantitative program evaluation measures will map onto the RE-AIM framework [83] (Table 3) and qualitative evaluation will map onto the i-PARIHS framework (Table 1).

**Table 3:** Quantitative program evaluation measures mapped onto the RE-AIM framework

RE-AIM domain	Evaluation measure
Reach	Patient enrollment, clinician referral counts
Effectiveness	Adherence, functional status
Adoption	Competency/fidelity among site mental health interventionists, CAE intervention engagement and attendance
Implementation	Intervention attendance, retention
Maintenance	Clinical site outpatient visits, no-show rate

##### Clinician referrals, health resource use

Prescribing providers will be encouraged to refer patients with BD that they believe are sub-optimally adherent and/or who would benefit from CAE. Referral sources and counts will be identified for all patients screened. To provide an indication of potential future maintenance/sustainability of the CAE intervention, resource use at both the clinical sites (outpatient visits, routine visit no-show rate) and off-site (emergency room visits and hospitalizations) will be evaluated. Self-reported resource use in the 6-month period prior to study enrollment and in the 12-month study period will be evaluated. To validate the self-report data, medical record documentation of service use will be evaluated in a randomly selected subset (20%) of the enrolled sample.

##### Patient qualitative evaluation

All participants in CAE will be asked to identify the degree to which the intervention met or did not meet their needs to help improve and maintain adherence. In addition, roadblocks as well as factors that can help use of CAE will be evaluated via qualitative interviews of patients who attended all or most (4 or 5) CAE sessions (*N*=10) and patients who attended 3 or fewer CAE sessions (*N*=10). The qualitative interview guide that was used to characterize participant perceptions in the CAE efficacy trial will be adapted for this effectiveness study. The interview guide will also be structured to align with i-PARIHS domains. Patient interviews will be conducted at the conclusion of the CAE modules and again at the 12-month follow-up point. To ensure a representative sample, we will attempt to balance by sex, race/ethnicity, and age and conduct the first set of patient interviews split equally in years 2 and 3 of the proposed project. All interviews will be recorded and transcribed.

##### Clinician, health system, SAB qualitative evaluation

At the conclusion of the modules, and again at the 12-month follow-up, prescribing clinicians (whether they referred an individual or not) will be queried regarding their perception of the effect of the intervention on patient adherence. In year 4, in order to optimize potential exposure to CAE in the clinic, interviews of a subset of providers representative of the mix of clinicians that provide care for patients with BD at the CMHC (*N*=10) will be conducted. All interviews will be recorded and transcribed.

## Data collection and management

Study data will be collected and managed using REDCap, a secure, web-based application designed to support data capture for research studies providing (1) an intuitive interface for validated data entry; (2) audit trails for tracking data manipulation and export procedures; (3) automated export procedures for seamless data downloads to common statistical packages; and (4) procedures for importing data from external sources [38]. Only study team members will be able to access the REDCap project which saves to servers in the CWRU Secure Research Environment. Data files, including analysis files, will be password protected to permit access and modification only by authorized persons. Participant names or similar potential identifiers (e.g., addresses, hospital record numbers) will not appear in any dataset.

Lists of potential participants will be saved within the secure server respective to the institution the patient is recruited from and will only be accessible by study staff, including CWRU research staff who have been research credentialed at both sites. The personal information will only be kept for as long as necessary (i.e., until the participant is enrolled or documented as a pre-screen failure). For those participants who cannot be contacted, refuse participation, or otherwise do not qualify for the study, only aggregate numbers will be retained to keep track of recruitment efforts. Careful attention will be given to confidentiality, which will be maintained using subject identification (ID) codes for enrolled participants. The list that links study ID codes with subject names and all forms bearing subject names and contact information will be stored in password-protected files on the respective institutions’ secure servers. Research files are not and will not be available to any unauthorized person.

As in the efficacy RCT, rigorous development of data collection forms and training of staff on the proper completion and checking of data collection forms will reduce errors at the point of collection. Additional data management practices before and after entry into the database will identify potential problems or outlying values and will catch other errors on data collection forms. Data management staff will be responsible for tracking forms entered and for performing routine auditing data checks. Analytic data sets will be prepared using SAS 9.2 or a similar statistical software package.

### Data analysis plan

For the primary intent-to-treat analyses (Aim 2), mixed effects longitudinal analysis of TRQ past week and past month over the 6 time periods will be conducted. The intention-to-treat sample will include all enrolled participants who complete baseline evaluation. A treatment variable will be included to indicate randomization to either CAE or eTAU. The lead trial statistician will be blinded for the analysis. Within the longitudinal model, significant interaction of the treatment variable with time indicates that the treatments have a different course of response. We will first fit models with time as a continuous covariate. Alternatively, we will also consider time period as a categorical variable. These interactions will be of primary interest. To account for possible imbalances across treatment groups and other sources of variation, explanatory variables such as sex and ethnicity and BD diagnosis type (I vs. II) will be considered for inclusion in the mixed models. Random effects will include a random intercept and possibly a random slope or other function of time as needed to suitably capture the within-person correlation structure. For TRQ, high success rates may lead to values concentrating on or close to the value zero in terms of percent medication missed, but there may be some persistent non-adherence as well, leading to non-normal data. With prior study data, we have taken the difference in subsequent TRQ scores from the baseline TRQ score to obtain resultant data that appear normally distributed. This is the approach we adopt for the power analysis below. As appropriate, we will also consider representing scores as binary outcomes, indicating whether or not an adherence threshold has been met (e.g., 80% adherent). We will thus consider generalized linear mixed models for binary outcomes (SAS PROC GLIMMIX), as well as fitting a longitudinal model on the difference values with baseline value adjustment. Graphical methods will be used extensively to examine distributions of residuals, identify potentially influential points, and guide data transformations to better approximate normality if warranted. Sensitivity analysis of results will be conducted by modeling a range of plausible correlation structures. Importantly, we will systematically assess the missing at random (MAR) assumption for mixed models. We will assess the sensitivity of the results through pattern mixture and selection model approaches that relax the missing at random assumptions [84].

We will study reasons for dropout and identify variables for possible inclusion in binary regression models of dropout by 10 weeks. Treatment differences in dropout models will be of interest. Additionally, we will compare corresponding TRQ and eCAP^TM^ adherence levels. Correlation between the measures will be estimated and Bland-Altman plots will be generated [85]. Secondary follow-up analyses at 9 and 12 months will also be compared in longitudinal analyses.

For Aim 3 and additional secondary analyses, GAF scores will be analyzed in a similar manner as in Aim 2. From previous experience, we expect GAF scores to approximately be normally distributed. Exploratory analysis will examine whether reductions in adherence barriers (e.g., BD knowledge, medication routines, communication, and substance) and increases in facilitators (patient satisfaction, ease of access, engagement) mediate the treatment effects on adherence. Further, secondary analyses will assess the mediation effects on functioning through adherence improvement. We will conduct simple mediation analyses, as well as multiple mediator models, using approaches described by Mackinnon and colleagues [86] and Preacher and Hayes [87]. Potential confounder variables will be included as covariates, such as age, sex, ethnicity, health literacy, BD type I vs. II, and substance use comorbidity. Bootstrapping methods will be used to assess indirect effects [87]. We will also consider, as needed, use of generalized linear models through a mediation formula approach [88, 89] to estimate the direct effect of the treatment and indirect effects through each mediator as well as through the set of mediators. Variable selection methods such as lasso will be used when multiple mediators are considered simultaneously to reduce redundancy. Moderators of treatment to be explored for each of the primary outcome models include covariates such as age, sex, ethnicity, health literacy, BD type I vs. II, and substance use comorbidity.

### Power analysis

Sample size projections are based on computations from the Repeated Measures and Sample Size (RMASS) software [90] with inputs estimated from prior study data. For sample size requirement for Aim 2, we used past-week TRQ as an approximation of expected and longer-term maintenance adherence status, and we consider primary outcomes to be at 6 months. First, note that at 6-month follow-up, there was a 34.6% improvement in TRQ observed with the CAE pilot, compared to 25.5% in the prior control arm (EDU). The control comparator used in the effectiveness trial, eTAU, is less intensive, with no extra face-to-face sessions, in contrast with EDU. For the control arm in a previous BD RCT [91], a 7% improvement in self-reported adherence was reported. The average of these differences is around 18%. Also, to alleviate deviation from normality, we model difference values from baseline, adjusting for the baseline value as a covariate. We note that compound symmetry and autoregressive (AR(1)) covariance models led to similar model fits in terms of treatment effect *p*-values and model fit criteria. Finally, in the previous CAE R01 study, we observed approximately 20% attrition by 6 months. We thus believe it is conservative to assume the following: (1) There is a mean difference of 16% in improvement from baseline at 6 months between CAE and eTAU; (2) under an error correlation structure of compound symmetry, suppose a small within-subject correlation near 0.08 and an error variance of 504, as estimated value from the data; and (3) assume that attrition will be 30% by 6 months. For a two-sided test of the treatment by time interaction effect being equal to zero, with alpha = 0.025 and power = 0.80, the required total sample size is approximately 182 in total.

Sample size requirement analyses were also conducted for GAF, the outcome measure for Aim 3. Again, inputs are based on estimates from the prior CAE R01 study. The observed mean increase in the CAE arm over 6 months was 6.1, and the difference between CAE and EDU was 3.5. In other BD RCTs conducted by this study team [91], little to no difference was observed from controls in GAF. As noted above, eTAU will be less intensive. Thus, we assume (1) a mean difference value of 4.8; (2) error variance of 48.3 with compound symmetry correlation of 0.04; and (3) 30% attrition at 6 months. For two-sided Type I error of 0.025, we estimate that 180 subjects will achieve power = 0.80. Thus, overall, for simultaneous Type I error between tests for Aims 1 and 2 to be at most 0.05, for power = 0.80 for each test, and to allow for some deviation from the assumptions used in the above power calculations, we will recruit a total of 190 subjects.

### Qualitative data analysis

In qualitative research, data collection, coding, and analysis occur simultaneously rather than sequentially. Emerging insights can be incorporated into later stages of data generation, enhancing the comprehensiveness of the results [92]. Transcript-based analysis [93, 94] will be used to analyze all qualitative data. In this method, the researcher uses the transcription itself as the source of the textural data to be analyzed. We will use a thematic content analysis approach to data analysis, encompassing open, axial and sequential coding, and the constant comparative method to generate constructs (themes) and elaborate the relationship among constructs [93, 94]. A coding dictionary that includes mutually exclusive code definitions will then be constructed. The coding structure will be reviewed after a preliminary analysis of a sub-sample of transcripts, and the dictionary will be refined through comparison, categorization, and discussion of each code’s properties and dimensions [93, 95].

### Provisions to monitor the data to ensure the safety of research participants

The data and safety monitoring plan for this project consists of three components, as outlined below.

#### Component 1

Approval from the local IRB will be obtained prior to performing any research related to this study, and approval will be maintained throughout the study period via continuing review.

#### Component 2

The project principal investigators (PIs) will review safety on an ongoing basis. The PI will meet regularly with staff (weekly or as necessary) to review any and all adverse events and to review the study data collected since the previous meeting. In addition, there are several committees within the larger study team that are involved in oversight of trial conduct and monitoring. These include the Study Coordinating Committee which is co-chaired by the study PIs, project manager, and research coordinators. The Study Coordinating Committee will be convened prior to the initiation of the project and will meet at regular intervals throughout the project. Meetings will be held in person or via videoconference. Additional meetings may be scheduled as necessary. Minutes of all meetings will be kept for reference and distributed to other relevant members of the study team as appropriate. A Clinical Trial Coordination Subcommittee will oversee patient recruitment, enrollment, assignment to treatment arm, scheduling of assessments, and patient flow through the trial. This subcommittee is chaired by study PIs, who will supervise the research assistant devoted to recruiting and patient enrollment. The Data Management Subcommittee is tasked with ensuring accurate data collection related to patient recruiting, enrollment, assessments, and interventions. This committee is chaired by the senior statistician lead who supervises the data management staff. The Data Management Subcommittee will ensure data integrity, completeness, and accuracy will be verified on an ongoing basis by this committee.

#### Component 3

The Data Safety and Monitoring Board (DSMB) will be comprised of a faculty member in the School of Medicine Department of Psychiatry who has expertise in BD, a faculty member who has expertise in the delivery of care in community mental health clinic settings, and a biostatistician. None of the DSMB will be part of the study team or affiliated with the trial sponsor, but all will have extensive experience with federally funded research and/or behavioral interventions. The DSMB will provide oversight of the proposed study via regular reports submitted to the DSMB by the PIs (minimally on an annual basis), telephone or email communication for issues that need more immediate attention, and ad hoc face-to-face meetings that might be called to evaluate unanticipated serious adverse events.

For this no-more-than-minimal risk behavioral study, adverse event reporting will be based on spontaneous reporting from study participants. Adverse events will be identified by the study investigators and/or qualified research assistants. All adverse events, whether considered serious or not, will be recorded and reviewed by the study PI on an ongoing basis, and reported to the IRB according to local IRB policy. Serious adverse events are defined as events that result in any of the following: death; a life-threatening experience; inpatient hospitalization or prolongation of existing hospitalization; a persistent or significant disability/incapacity; or a congenital anomaly/birth defect (or an event that may require medical or surgical intervention to prevent one of the outcomes listed above). Given that this is deemed a minimal risk trial, there will be no interim analyses or stopping guidelines.

All participants will continue with their psychiatric providers during and following the trial. Any participant at risk for imminent harm to self or others will be handled on a case-by-case basis. Participants will be terminated from the trial if deemed unsafe to continue in study procedures by the PI or the participant’s clinician (such as for worsening condition or increased harm risk), if the participant requests to be removed or withdraws consent, or if the participant is lost to follow-up. Since the intervention is an addition to their regular treatment and does not include medication, there will be no follow-up with the research team. Should the need arise to discontinue a patient from the study because of symptom worsening or for any other reason, we will collaborate with their non-study-related clinician to determine treatment options for that patient following study discontinuation. There will be no post-trial care or compensation for those who suffer harm from trial participation.

### Dissemination

Following completion of the last trial participant, results regarding the primary outcomes of the trial will be submitted for publication within 1 year of the final study visit. Authorship for all future trial publications will require that the individual will have substantially contributed to the work, including conception, methodology, data acquisition, analysis, and interpretation. We will use International Committee of Medical Journal Editors (ICMJE) criteria to determine authorship [96].

Completed study data for public access will be uploaded to the U.S. National Institute of Mental Health (NIMH) National Data Archive (NDA). All final peer-reviewed manuscripts that arise from this proposal will be submitted to the digital archive PubMed Central. The results generated from this grant will be presented at national or international conferences and published in a timely fashion in journals in the field of psychopharmacology, behavioral interventions, and care and service delivery to people with chronic and persistent serious mental illness. Similarly, results from the trial will be presented at conferences focused on bipolar disorder and public mental health care. In addition to making study findings available to the broader scientific community, the study team will share results with the SAB assisting in the refinement of the CAE intervention and integrating CAE into clinical site workflow. The study team will also hold several in-service presentations targeted to clinical and administrative staff at the CMHC and safety-net clinical sites who may not read journal articles or attend scientific meetings.

## Discussion

The proposed project addresses a problem with enormous consequences—medication treatment non-adherence. Among individuals with BD, treatment non-adherence imposes an extensive burden and is associated with high social and economic costs. An emerging literature suggests that adherence enhancement might work best by addressing factors that are important and modifiable for a specific individual [1, 34]. By focusing on a high-risk, vulnerable group with BD, and refining an evidence-based approach that will integrate into workflow of public-sector care/community mental health clinics, the potential for improving BD adherence on a broad level is substantial. In summary, the proposed work is significant in that it addresses a major public health need—interventions that improve treatment adherence for people with BD. The proposed study is designed to specifically target non-adherent individuals with BD and addresses the need for practical interventions that are effective, flexible, and designed to adapt to different settings and patients.

## Trial status

We used CAE-E protocol from September 2, 2021, and anticipate starting recruitment for the clinical trial in October of 2021 and completing recruitment by the end of 2024.

## Supplementary Information

**Additional file 1.**

**Additional file 2.**

## Data Availability

The authors of this paper at CWRU will have full access to the final trial dataset.

## Abbreviations

BD: Bipolar disorder
CAE: Customized adherence enhancement
RCT: Randomized controlled trial
SAB: Stakeholder advisory board
CMHC: Community mental health clinic
i-PARIHS: Integrated Promoting Action on Research Implementation in Health Services
eTAU: Enhanced treatment as usual
TRQ: Tablet Routines Questionnaire
IRB: Institutional Review Board
REDCap: Research Electronic Data Capture
AMSQ: Attitudes towards Mood Stabilizers Questionnaire
ROMI: Rating of Medication Influences
MET: Modified Motivational Enhancement Therapy
MITI: Motivational Interviewing Treatment Integrity
MEMS: Medication Event Monitoring System
REALM-R: Rapid Estimate of Adult Literacy in Medicine Revised
OBQ: Oxford Bipolar knowledge Questionnaire
SRHI: Self-Report Habit Index
AUDIT: Alcohol Use Disorders Identification Test
DAST: Drug Abuse Screening Test
SOCRATES: Stages of Change Readiness and Treatment Eagerness Scale
SES: Service Engagement Scale
CSQ: Client Satisfaction Questionnaire
GAF: Global Assessment of Functioning
BPRS: Brief Psychiatric Rating Scale
YMRS: Young Mania Rating Scale
MADRS: Montgomery Asberg Depression Rating Scale
CGI: Clinical Global Impressions
MAR: Missing at random
RMASS: Repeated Measures and Sample Size
AR(1): Autoregressive
PI: Principal Investigator
DSMB: Data Safety and Monitoring Board
