# Supplementary material for: A randomized controlled trial of customized adherence enhancement (CAE-E): study protocol for a hybrid effectiveness-implementation project
Source: Trials. 2022 Aug 4;23:634. doi: 10.1186/s13063-022-06517-0 (PMC9351150; doi:10.1186/s13063-022-06517-0)
Supplement: Supplementary file 1 — Additional file 1. [file 13063_2022_6517_MOESM1_ESM.pdf]

CASE WESTERN RESERVE UNIVERSITY EST. 1826

INFORMED CONSENT DOCUMENT

Effectiveness RCT of Customized Adherence Enhancement (CAE-E) – SAB member

You are being asked to participate in a research study conducted by researchers at Case Western Reserve University. This consent form contains important information about this project and what to expect if you decide to participate. Please consider the information carefully. Feel free to ask questions before making your decision whether or not to participate. Your participation in this research is voluntary.

KEY INFORMATION FOR YOU TO CONSIDER:

The following is a short summary of this study to help you decide whether or not to be a part of this study. More detailed information is listed later on in this form.

Purpose

The purpose of this research is to convene a Stakeholder Advisory Board (SAB) to assist the researchers in refining the Customized Adherence Enhancement (CAE) intervention, an educational and behavioral program, helps patients to take their medications and reduce the number of missed doses, compared to receiving information and reminders through texts or phone calls.

Procedures and Duration

We expect that you will be in this research study for about 5 years.
During that time you will be asked to be part of several video-conferencing meetings that will focus on helping the researchers to refine the CAE intervention.
Please refer to the Detailed Consent for a complete description of the study procedures.

Reasons You Might Choose to Volunteer For This Study

This study may or may not benefit you, but you may find it helpful to share your experiences with others. Please refer to the Detailed Consent for a complete description of the anticipated benefits.

Reasons You Might Choose Not to Volunteer For This Study

It is possible that some of the questions you are asked may be upsetting, or you may feel uncomfortable answering them. If you do not wish to answer a question, you may skip it and go to the next question.
Please refer to the Detailed Consent for a complete description of the foreseeable risks and discomforts.

Voluntary Participation:

If you decide to participate in the research, it should be because you want to volunteer. There is no penalty or loss of benefits for not participating or for discontinuing your participation.
Please refer to the Detailed Consent for additional information.

IRB NUMBER: STUDY20210857
IRB APPROVAL DATE: 8/26/2021
IRB EFFECTIVE DATE: 9/17/2021
IRB EXPIRATION DATE: None

DETAILED CONSENT

You were selected as a possible participant because you receive care for bipolar disorder from MetroHealth or The Nord Center *or* you are a family member of an individual with bipolar disorder *or* you are a clinician or administrator who practice at a safety-net care system or a Community Mental Health Clinic (CMHC). We hope to recruit 12 people to volunteer for this part of the research study.

Procedures

Your participation in this study will last for 4-5 years and will involve 12 video-conference meetings.
The SAB will be composed of up to 12 relevant stakeholders including 4 individuals with BD who receive their care in safety-net care systems and/or a CMHC, 2 family members of individuals with BD, 4 clinicians, and 2 administrators who practice in these locations.

If you agree to participate in this research, we would ask you to do the following things:

1. sign the informed consent form (10-20 minutes).
2. participate in 3 advisory board teleconference meetings within the first 6 months of the project to help researchers refine CAE to use in a safety-net or CMHC (about 60-90 minutes each meeting). In the first meeting, SAB members will review the CAE curriculum and identify content areas that they feel may need to edited or added. The study team members will then make these modifications to the CAE intervention manual. In the 2nd meeting, SAB members will review the revised CAE content and make any additional /final suggestions. In the 3rd SAB meeting, the SAB will be asked to identify strategies that will be helpful to integrating CAE into clinic workflow.
3. participate in 2 advisory board teleconference meetings per year for the rest of the study to advise the researchers on the most effective way to implement the project in the community. It is expected that during these follow-up meetings, SAB input and guidance will continue to assist with optimal implementation and effectiveness of the project include maximizing recruitment and retention of participants and clinician engagement and referrals.
4. Answer some basic demographic and information about experience with bipolar disorder through a survey emailed to you or directly to a research assistant who will record your answers.

You will be video/audio recorded while you are in the advisory board meetings.

Foreseeable Risks and Discomforts

All treatments and procedures may involve some level of risk to you.

Any time information is collected, there is a potential risk for loss of confidentiality. There are no other known risks of harms or discomforts associated with this study beyond those encountered in normal daily life. Some of the activities we will ask you to complete might make you feel uncomfortable or tired. You may refuse to answer any of the questions, take a break, or stop your participation in this study at any time.

Anticipated Benefits

You will not directly benefit from participation in this study.

You may find it helpful to talk about your experiences with others. Information obtained in this study may help improve care for other patients with bipolar disorder.

Compensation

There will be no costs to you for study participation.

You will receive the following compensation/reimbursement:
You will receive $30 for each SAB meeting you attend. Total compensation for participation in this study is $360.

IRB NUMBER: STUDY20210857
IRB APPROVAL DATE: 8/26/2021
IRB EFFECTIVE DATE: 9/17/2021
IRB EXPIRATION DATE: None

The Accounting Department at Case Western Reserve University will be given your name, address, and Social Security Number in order to process payment for your study participation. Study payments are considered taxable income and reportable to the IRS. A Form 1099 will be sent to you if your total payments are $600 or more in a calendar year.

Alternative(s) to Participation

You have the option to not participate.

Voluntary Nature of the Study

Your participation is voluntary. If you choose not to participate, it will not affect your current or future relations with the University, MetroHealth, or The Nord Center. There is no penalty or loss of benefits for not participating or for discontinuing your participation.

You are free to withdraw from this study at any time. If you decide to withdraw from this study, you should notify the research team immediately. The research team may also end your participation in this study if you do not follow instructions, miss scheduled visits, or if your safety or welfare are at risk.

If you withdraw or are removed from the study, the researcher may ask you to return for a final visit or evaluation, but you may choose not to participate in these activities.

Confidentiality

Every effort will be made to keep your information confidential; however, this cannot be guaranteed.
As a member of the SAB we ask that you respect the privacy of the other members and not share information discussed during meetings with others outside of the study.

Research records will be kept in a secure location and access will be limited to the researchers, the University review board responsible for protecting human participants, and regulatory agencies. In any sort of report we might publish, we will not include any information that will make it possible to identify a participant.

Certificate of Confidentiality

This research is covered by a Certificate of Confidentiality (CoC) from the National Institutes of Health. This means that we will not tell anyone what you tell us even if a judge tries to force us to identify you as a person in the study unless you give us permission. You should know, however, that we may tell local authorities if harm to you, harm to others, or if child abuse or neglect becomes a concern or if we are required by federal, state or local law. Also, the government agency that has provided the money for this project may see your information if they ask for our records to ensure we were conducting the project correctly. In addition, we may use certain information in future research as permitted by law.

Subject Identifiable Information

All information that identifies you will be removed from the study data and replaced with a code. A list linking the code and your identifiable information will be kept separate from the research data. The only personal identifiers that will be retained will be used to contact you for future appointments and to pay your stipends.

The audio/video recordings that can identify you will be transcribed and deleted at the end of the study.

Data Retention

The researchers intend to keep the research data for approximately 5 years after the end of the research study.

Contacts and Questions

The researchers conducting this study are Martha Sajatovic MD, Jennifer Levin PhD, Carol Blixen PhD, Douglas Einstadter MD, and Farren Briggs PhD You may ask any questions you have now. If you have any additional questions, concerns or complaints about the study, you may contact the researchers at 216-844-2400.

If you would like to talk to someone other than the researchers about questions or complaints regarding this study, research participant rights, research-related injuries, or other concerns, please contact:
Case Western Reserve University Institutional Review Board
10900 Euclid Ave.
Cleveland, OH 44106-7230
(216) 368-4514

Statement of Consent

Your signature below certifies the following:

- You are at least 18 years of age.
- You have read (or been read) the information provided above.
- You have received answers to all of your questions and have been told who to call if you have any more questions.
- You have freely decided to participate in this research.
- You understand that you are not giving up any of your legal rights.

You will be given a copy of this form for your records.

____
Printed Name of Participant

____ Date: ___________
Signature of Participant

____ Date: ___________
Signature of Person Obtaining Consent
