# Supplementary material for: A randomized controlled trial of customized adherence enhancement (CAE-E): study protocol for a hybrid effectiveness-implementation project
Source: Trials. 2022 Aug 4;23:634. doi: 10.1186/s13063-022-06517-0 (PMC9351150; doi:10.1186/s13063-022-06517-0)
Supplement: Supplementary file 2 — Additional file 2. [file 13063_2022_6517_MOESM2_ESM.pdf]

CASE WESTERN RESERVE UNIVERSITY EST. 1826

INFORMED CONSENT DOCUMENT

Effectiveness RCT of Customized Adherence Enhancement (CAE-E) – RCT participant

You are being asked to participate in a research study conducted by researchers at Case Western Reserve University. This consent form contains important information about this project and what to expect if you decide to participate. Please consider the information carefully. Feel free to ask questions before making your decision whether or not to participate. Your participation in this research is voluntary.

KEY INFORMATION FOR YOU TO CONSIDER:

The following is a short summary of this study to help you decide whether or not to be a part of this study. More detailed information is listed later on in this form.

Purpose

The purpose of this research is to find out if Customized Adherence Enhancement (CAE), an educational and behavioral program, helps patients to take their medications and reduce the number of missed doses, compared to enhanced Treatment As Usual (eTAU) of receiving information and reminders through texts or phone calls.

Procedures and Duration

We expect that you will be in this research study for about 12 months. During that time you will be asked to attend 6 study assessment visits and receive monthly information and reminders by text or phone call. If you are assigned to the CAE intervention group you will also have 2 in-person sessions, 3 video-conferencing or phone education sessions, and 1 brief phone call.

In-person visits will be conducted at the Department of Psychiatry at MetroHealth or at the Nord Center depending on which site you receive your mental health care at. All of the assessment visits and sessions can be done by video-conferencing or phone if an in-person visit is not possible.

Please refer to the Detailed Consent for a complete description of the study procedures.

Reasons You Might Choose to Volunteer For This Study

This study may or may not benefit you, but you may find it helpful to participate in the educational sessions. Please refer to the Detailed Consent for a complete description of the anticipated benefits.

Reasons You Might Choose Not to Volunteer For This Study

It is possible that some of the questions you are asked may be upsetting, or you may feel uncomfortable answering them. If you do not wish to answer a question, you may skip it and go to the next question. You may also feel tired after completing all the questionnaires or the educational sessions.

Please refer to the Detailed Consent for a complete description of the foreseeable risks and discomforts.

Voluntary Participation:

If you decide to participate in the research, it should be because you want to volunteer. There is no penalty or loss of benefits for not participating or for discontinuing your participation.

Please refer to the Detailed Consent for additional information.

DETAILED CONSENT

You were selected as a possible participant because you receive care for bipolar disorder at a mental health clinic and you have missed some of your bipolar medication doses in the past week or month. We hope to recruit One hundred and ninety (190) people to volunteer for this research.

Procedures

Frequency of Visits

We expect that you will be in this research study for about 12 months. During that time you will be asked to attend 6 study assessment visits and receive monthly information and reminders by text or phone call. If you are assigned to the CAE intervention group you will also have 2 in-person session, 3 video-conferencing or phone education sessions, and 1 brief phone call.

In-person visits will be conducted at the Department of Psychiatry at MetroHealth or at the Nord Center depending on which site you receive your mental health care at. All of the assessment visits and sessions can be done by video-conferencing or phone if an in-person visit is not possible.

The study visits include:

Screening

At this visit, the following screening procedures will be performed to determine if you can take part in this study:

- you will sign the informed consent form;
- you will receive a psychiatric diagnostic assessment;
- You will be asked basic demographic questions about facts such as your age and gender;
- You will be asked questions about your psychiatric symptoms and how you are taking your medication; and
- You will be given a special cap called an eCap to place on one of your pill bottles. You will be asked to bring the bottle with the eCap on it at each visit or download a free app to your Smartphone so you can scan the eCap at the time of your assessment visit.

The screening visit will last between 120 minutes or 2 hours and 180 minutes or 3 hours.
If you fit all study criteria after completing your screening visit, you will be asked to return in 1 to 2 weeks for a baseline visit.

Baseline

Your baseline visit will be completed 1 to 2 weeks after your screen visit. At the baseline visit, you will be asked questions about your psychiatric symptoms, attitudes, how you are taking your medication, how well you are able to function in your life, and what healthcare services you have used recently.

You will be randomized to either the CAE (experimental) or eTAU (control) intervention (see below for intervention descriptions).
The baseline visit will last about 90 minutes.

Randomization/Study Intervention

If you participate in this study, you will be assigned to a study group by chance using a process similar to the flip of a coin. This process is called randomization. Neither you nor study staff will select the group to which you will be assigned. The study staff will let you know which group you are in.

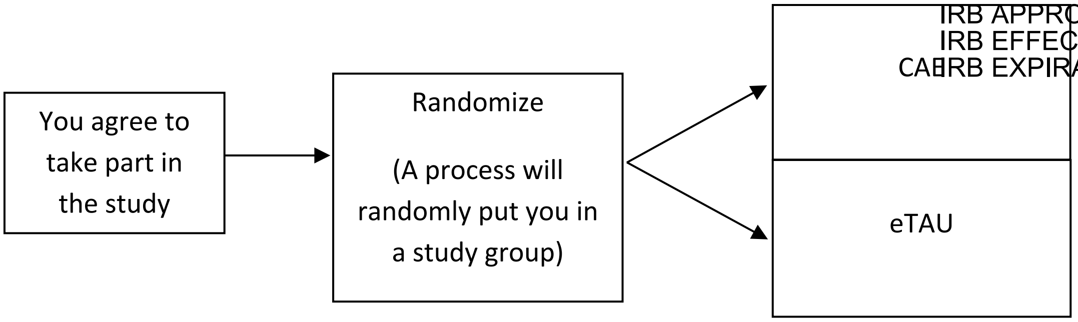

<u>Experimental Intervention Sessions (CAE):</u>
If you are randomized to the CAE group, you will begin the intervention within a week after your Baseline visit.

There will be 1 in-person session and 3 video teleconference or phone sessions over 4-6 weeks. During those sessions you will meet with a member of the study team and you will receive the study's educational and behavioral intervention (CAE). During the session, you will receive information on how to deal with your condition and take your medications, and will be able to ask questions. About 4 weeks after the fourth session you will have an additional in-person session. Between the fourth and final session you will receive a phone call check-in.

If it is not possible to have the first and last visits in-person, they can be done by video teleconference or by phone. The sessions will be recorded and some recordings will be randomly selected and watched by the researchers to make sure the information in the sessions is being given in a consistent way.

During the time between your Baseline assessment and your 12-month assessment you will also receive monthly automated text messages (or phone calls if you prefer not to receive texts) to refill medications, fill eCAPs and brief adherence messages.

Each session will last about 60 minutes.

<u>Control Intervention Sessions (eTAU):</u>
If you are randomized to the eTAU group you will also begin the intervention within a week after your Baseline visit. You will receive monthly automated text messages (or phone calls if you prefer not to receive texts) to refill medications, fill eCAPs, and brief adherence messages.

No matter which group you are randomized to, you will still continue with the regular care you get from your usual provider throughout your participation in the study.

Follow-up assessment visits
At 10 weeks, 6 months, 9 months, and 12 months after your baseline visit you will be asked questions about your attitudes, psychiatric symptoms, how you are taking your medication, how well you are able to function in your life, and what healthcare services you have used recently.
Each of these visits will last about 90 minutes.

Some of the questionnaires from the Screening, Baseline, and Follow-up assessment visits may be emailed to you as a survey. If you are not able to or prefer not to complete the survey on your own the research assistant can ask you the questions during your assessment visit instead.

In addition to the above, we may also send a short survey to your provider at 10 weeks and 12 months to get their input on how you are doing with your treatment adherence.

Optional Qualitative Interviews
In addition to the study visits and educational intervention, if you are randomized to CAE, you may be asked to participate in two additional interviews, one within a couple of days of your fourth CAE session and one on or within a couple of days of your 12-month assessment. During these interviews you will be asked questions about topics including your illness, how you experience your illness, how you communicate with others and your doctor, and how you are doing in the study.

These interviews will be recorded. Participation in these qualitative interviews is voluntary and you can decide whether or not you want to participate. You do not have to participate in the qualitative interviews to be able to participate in the main study.

Depending on when you are starting this study, we may already have enough people who have agreed to participate in the additional interviews. If we are no longer accepting people into the additional interviews, we will tell you.

Please indicate your choice by checking one of the boxes below:

- ☐ Yes, I am willing to participate in the additional interviews if the researchers ask me to and I understand that these interviews will be recorded.
- ☐ No, I am not willing to participate in the additional interviews and I understand that I may still participate in the main part of the study.
- ☐ I have been informed that the study is not open for this part.

You can choose to stop participating for any reason at any time. However, if you decide to stop participating in the study, we encourage you to tell the researchers. You may be asked to complete some study termination activities, but you may choose not to participate in these activities.

Foreseeable Risks and Discomforts
All treatments and procedures may involve some level of risk to you.

Any time information is collected, there is a potential risk for loss of confidentiality. There are no other known risks of harms or discomforts associated with this study beyond those encountered in normal daily life. Some of the activities we will ask you to complete might make you feel uncomfortable or tired. You may refuse to answer any of the questions, take a break, or stop your participation in this study at any time.

Anticipated Benefits
You will not directly benefit from participation in this study.
You may find it helpful to participate in the educational sessions and/or the text/phone messages. It is possible that the interventions may help you remember to take your medication. Information obtained in this study may help improve care for other patients with bipolar disorder.

Compensation
While there are no direct costs to being in the study, by participating you may need to find childcare, take off work, or find transportation.

You will receive assistance with transportation to each of the in-person assessment and intervention visits in the form of a bus pass or parking pass.

You will receive the following compensation/reimbursement:
You will receive $30 for each of the first 5 assessment visits you complete and $40 for the final, 12 month assessment visit. You will receive an additional $10 for each visit to which you bring the eCAP or sync it with the smartphone app.
Total compensation for participation in this study is $240.

If you decide to withdraw from the study or are withdrawn by the research team, you will receive compensation for the visits that you have completed.

If you participate in the optional qualitative interviews you will receive $25 per interview completed. Total compensation for participation in the optional qualitative interview is $50.

The Accounting Department at Case Western Reserve University will be given your name, address, and Social Security Number in order to process payment for your study participation. Study payments are considered taxable income and reportable to the IRS. A Form 1099 will be sent to you if your total payments are $600 or more in a calendar year.

Alternative(s) to Participation

You have the option to not participate.

Voluntary Nature of the Study

Your participation is voluntary. If you choose not to participate, it will not affect your current or future relations with the University, MetroHealth, or The Nord Center. There is no penalty or loss of benefits for not participating or for discontinuing your participation.

You are free to withdraw from this study at any time. If you decide to withdraw from this study, you should notify the research team immediately. The research team may also end your participation in this study if you do not follow instructions, miss scheduled visits, or if your safety or welfare are at risk.

If you withdraw or are removed from the study, the researcher may ask you to return for a final visit or evaluation, but you may choose not to participate in these activities.

If you elect to withdraw or are withdrawn from this research study, you may choose to terminate the continued use or disclosure of your protected health information (PHI) for research purposes. The request to end the use or disclosure of your PHI must be made in writing to the Principal Investigator. If the data collected are de-identified (anonymous) the researchers will not have the ability to remove your study data.

Privacy of Protected Health Information (PHI)

The Health Insurance Portability & Accountability Act (HIPAA) is a Federal law that helps to protect the privacy of your health information and to whom this information may be shared within and outside of Case Western Reserve University, MetroHealth, and The Nord Center. This Authorization form is specifically for a research study entitled "Effectiveness RCT of Customized Adherence Enhancement (CAE-E)" and will tell you what health information (called Protected Health Information or PHI) will be collected for this research study, who will see your PHI and in what ways they can use the information. In order for the Principal Investigators, Dr. Martha Sajatovic and Dr. Jennifer Levin, and the research study staff to collect and use your PHI, you must sign this authorization form. You will receive a copy of this signed Authorization for your records. If you do not sign this form, you may not join this study. Your decision to allow the use and disclosure of your PHI is voluntary and will have no impact on your treatment at MetroHealth or The Nord Center. By signing this form, you are allowing the researchers for this study to use and disclose your PHI in the manner described below.

Generally, the Principal Investigator and study staff at Case Western Reserve University, MetroHealth, and The Nord Center who are working on this research project will know that you are in a research study and they will see and use your PHI. The researchers working on this study will collect the following PHI about you:

- your name, initials, address, telephone number, date of birth and other demographic information;
- your medical history (including the history and diagnosis of your disease and your family medical history) and the name of your physician(s) and locations where you received any treatment;

- information about other medical conditions that may affect your participation, including information relating to mental health, behavioral health and psychiatric disorders; and alcohol and drug dependence or abuse;
- specific information about any treatment/therapy you receive while participating in the research study and treatment you received prior to the research study (including treatments and therapies, surgeries, hospitalizations and medications);
- information about how frequently you take your medications;
- information about your general health status and the status of your disease or medical condition; and
- numbers or codes that identify you such as your social security number, medical record number, and research study case number.

This PHI will be used to determine if an educational and behavioral intervention program is helpful in the treatment of patients with bipolar disorders. Your access to your PHI may be limited during the study to protect the study results.

Your PHI may also be shared with the following groups/persons associated with this research study or involved in the review of research: Case Western Reserve University, including staff from the Department of Psychiatry and Department of Neurology; other staff from the Principal Investigator's medical practice group; staff at MetroHealth or The Nord Center; Government representatives or Federal agencies, when required by law.

Your permission to use and disclose your PHI does not expire. However, you have the right to change your mind at any time and revoke your authorization. If you revoke your authorization, the researchers will continue to use the information that they previously collected, but they will not collect any additional information. Also, if you revoke your authorization you may no longer be able to participate in the research study. To revoke your permission, you must do so in writing by sending a letter to Martha Sajatovic, M.D., Department of Psychiatry – 7th floor, Case Western Reserve University, 10524 Euclid Ave., Cleveland, OH 44106.
If you have a complaint or concerns about the privacy of your health information, you may also write to the University's Director of Privacy Management, Lisa Palazzo at lisa.palazzo@case.edu or 216-368-4286 or to the Federal Department of Health and Human Services (DHHS) at DHHS Regional Manager, Office for Civil Rights, US Department of Health and Human Services, 233 N. Michigan Ave., Suite 240, Chicago, IL 60601.

The researchers and staff agree to protect your health information by using and disclosing it only as permitted by you in this Authorization and as directed by state and Federal law. Case Western Reserve University, MetroHealth, and The Nord Center are committed to protecting your confidentiality. Please understand that once your PHI has been disclosed to anyone outside of, Case Western Reserve University, MetroHealth, and The Nord Center, there is a risk that your PHI may no longer be protected.

Confidentiality

Every effort will be made to keep your information confidential; however, this cannot be guaranteed. Research records will be kept in a secure location and access will be limited to the researchers, the University review board responsible for protecting human participants, and regulatory agencies. In any sort of report we might publish, we will not include any information that will make it possible to identify a participant. The information about you that is collected in this study will be shared with the study sponsor and may be combined with information gathered from public sources or other research studies. This information may be used for purposes unrelated to this research and could potentially be used to identify you.

However, you should understand that in cases where we suspect elder or child abuse or neglect or imminent harm to self or others, we will take the necessary action in an effort to prevent such harm or injury, including reporting to authorities.

Additionally, if you are in danger of hurting yourself or others, the study team may reach out to your mental health provider for assistance.

You have been given a copy of the privacy policy from the eCAP company. By signing this consent you are also acknowledging and agreeing to that privacy policy.

Certificate of Confidentiality

This research is covered by a Certificate of Confidentiality (CoC) from the National Institutes of Health. This means that we will not tell anyone what you tell us even if a judge tries to force us to identify you as a person in the study unless you give us permission. You should know, however, that we may tell local authorities if harm to you, harm to others, or if child abuse or neglect becomes a concern or if we are required by federal, state or local law. Also, the government agency that has provided the money for this project may see your information if they ask for our records to ensure we were conducting the project correctly. In addition, we may use certain information in future research as permitted by law.

Subject Identifiable Information

All information that identifies you will be removed from the study data and replaced with a code. A list linking the code and your identifiable information will be kept separate from the research data. The only personal identifiers that will be retained will be used to contact you for future appointments and to pay your stipends.

The audio/video recordings that can identify you will be transcribed and deleted at the end of the study.

Data Retention

The researchers intend to keep the research data for approximately 5 years after the end of the research study.

Significant New Findings

If any significant new finding develop that may affect your decision to participate these will be provided to you.

Contacts and Questions

The researchers conducting this study are Martha Sajatovic MD, Jennifer Levin PhD, Carol Blixen PhD, Douglas Einstadter MD, and Farren Briggs PhD You may ask any questions you have now. If you have any additional questions, concerns or complaints about the study, you may contact the researchers at 216-844-2400.

If you would like to talk to someone other than the researchers about questions or complaints regarding this study, research participant rights, research-related injuries, or other concerns, please contact:
Case Western Reserve University Institutional Review Board
10900 Euclid Ave.
Cleveland, OH 44106-7230
(216) 368-4514

Additional Information

Should you find that you need additional assistance with mental health topics we are providing these hotlines for your reference. These organizations are not connected to this research study.
National Suicide Prevention Hotline (for anyone in suicidal crisis or emotional distress) 1-800-273-8255 (TALK)
National Domestic Violence Hotline -- 1-800-799-7233
National Deaf Domestic Violence Hotline -- 1-855-812-1001
Substance Abuse and Mental Health Services Administration (SAMHSA): 1-800- 662-4357
Crisis Text Line- Text HOME to 741741
RAINN (Rape, Abuse and Incest National Network) 1-800-656-4673 https://www.rainn.org/
To talk to someone part of the LGBTQIA+ community:
The Trevor Project -- 1-866-488-7386 or text START to 678678
Trans Lifeline -- 1-877-565-8860

Statement of Consent

Your signature below certifies the following:

- You are at least 18 years of age.
- You have read (or been read) the information provided above.
- You have received answers to all of your questions and have been told who to call if you have any more questions.
- You have freely decided to participate in this research.
- You understand that you are not giving up any of your legal rights.

You will be given a copy of this form for your records.

____
Printed Name of Participant

____ Date: ____________
Signature of Participant

____ Date: ____________
Signature of Person Obtaining Consent
